# A Scalable Video Multicast Scheme Based on User Demand Perception and D2D Communication

**DOI:** 10.3390/s23177325

**Published:** 2023-08-22

**Authors:** Ruiqi Ouyang, Xuanrui Xiong, Mingkai Fu, Jie Wang, Shixiong Chen, Osama Alfarraj

**Affiliations:** 1 School of Communication and Information Engineering, Chongqing University of Posts and Telecommunications, Chongqing 400065, China; 2 School of Software, Dalian University of Technology, Dalian 116024, China; 3 Computer Science Department, Community College, King Saud University, Riyadh 11437, Saudi Arabia

**Keywords:** D2D communication, scalable video, video recommendation, spectrum sharing, multicast

## Abstract

With the widespread application of 5G technology, there has been a significant surge in wireless video service demand and video traffic due to the proliferation of smart terminal devices and multimedia applications. However, the complexity of terminal devices, heterogeneous transmission channels, and the rapid growth of video traffic present new challenges for wireless network-based video applications. Although scalable video coding technology effectively improves video transmission efficiency in complex networks, traditional cellular base stations may struggle to handle video transmissions for all users simultaneously, particularly in large-scale networks. To tackle this issue, we propose a scalable video multicast scheme based on user demand perception and Device-to-Device (D2D) communication, aiming to enhance the D2D multicast network transmission performance of scalable videos in cellular D2D hybrid networks. Firstly, we analyze user interests by considering their video viewing history and factors like video popularity to determine their willingness for video pushing, thereby increasing the number of users receiving multicast clusters. Secondly, we design a cluster head selection algorithm that considers users’ channel quality, social parameters, and video quality requirements. Performance results demonstrate that the proposed scheme effectively attracts potential request users to join multicast clusters, increases the number of users in the clusters, and meets diverse user demands for video quality.

## 1. Introduction

With the remarkable advancement of wireless communication technology and the rapid evolution of online media content, intelligent wireless communication devices have achieved widespread proliferation, inducing a substantial metamorphosis in wireless network traffic patterns. The transition from voice-centric activities to the dominance of social media platforms, such as Weibo, WeChat, and QQ, alongside the emergence of network live streaming and short video content, has instigated an unprecedented upsurge in wireless data consumption. According to Cisco’s projections, the global data traffic is poised to escalate to 396EB per month by 2022, surpassing the cumulative data traffic from the inception of the internet until the close of 2016. Notably, the proliferation of voluminous data traffic is principally fueled by the realm of network video streaming, which is anticipated to encompass 17% of the total internet video traffic, significantly escalating from the previous 5%. Furthermore, the consumption of network live-streaming data is forecasted to undergo a fifteen-fold surge by 2022 [1]. This exponential surge in data traffic presents formidable challenges to conventional cellular networks, accentuating the scarcity of wireless resources [2].

In order to address the elevated demands for high bandwidth and low latency within wireless networks while simultaneously alleviating the strain on base stations, the fifth generation (5G) networks have introduced transformative objectives, namely Enhanced Mobile BroadBand (eMBB) and Ultra-Reliable Low Latency Connection (URLLC). Moreover, the significance of Device-to-Device (D2D) technology has been acknowledged as a pivotal approach for mitigating latency and reducing base station burdens. Particularly in contexts involving extensive data transmission and processing, such as those encountered in smart cities, D2D communication offers distinct advantages, encompassing elevated transmission rates and minimized latency. This renders it particularly suitable for transmitting substantial data payloads, including video content necessitating robust throughput [3]. Furthermore, D2D communication assumes heightened significance in settings characterized by infrastructure damage and constrained or even absent network resources, such as public safety zones, remote mountainous regions, or densely populated venues, like sports stadiums, during globally attended events, like the Olympics or World Cup. Amidst such periods of network congestion, individuals frequently stream identical real-time video content on their respective devices. Capitalizing on this scenario, D2D communication emerges as a viable solution to relay real-time video requests among proximate devices endowed with favorable channel quality and a pronounced willingness to participate [4].

In the domain of mobile communication networks, the scarcity of wireless spectrum and computing resources presents significant hurdles. Scalable video coding (SVC) technology emerges as a promising solution. SVC partitions a video into distinct constituents, including a fundamental base layer and multiple augmentation layers. These augmentation layers can be dynamically chosen in response to fluctuating channel conditions, thereby optimizing the judicious employment of precious channel resources. Prioritizing the transmission of the base layer ensures that fundamental communication requisites are fulfilled, even amid suboptimal channel circumstances, albeit with a compromise on video quality. Conversely, during favorable channel conditions, the selection of supplementary enhancement layers becomes viable, ushering in heightened frame rates and resolutions, consequently elevating the comprehensive video quality. By amalgamating video recommendation principles with the adaptive capabilities of scalable video transmission, we can effectively harness available resources, affording users an optimal viewing encounter meticulously tailored to their distinct preferences and the prevalent network conditions.

In summary, within the domain of scalable video transmission on the Internet of Things (IoT) facilitated by D2D communication, the utilization of intelligent methodologies for a comprehensive analysis encompassing user historical data, social parameters, channel quality, and other pertinent aspects of big data, offers substantial academic merit in augmenting the quality of diversified communication services. This undertaking assumes paramount importance in proficiently harnessing spectrum resources, mitigating base station burdens, and satisfying users’ requisites for elevated bandwidth and minimized latency.

This paper predominantly centers on investigating the matter of scalable video transmission within cellular D2D hybrid networks. The principal contributions of this study are outlined below:

1. Investigation of D2D Multicast for Scalable Videos: This research introduces a scalable video multicast approach centered around user demand perception for video dissemination. Leveraging insights from users’ historical video consumption patterns and capturing their preferences, this study offers intelligent video recommendations to potential requestors. Additionally, an innovative clustering algorithm is devised, which augments the user count within D2D multicast clusters.
2. Cluster Head Selection: A cluster head selection algorithm is formulated, taking into account inter-user channel quality, social parameters, and users’ preferences for video quality. This algorithm establishes a holistic metric aimed at optimizing multicast effectiveness, thereby achieving the highest possible efficiency in D2D multicast operations.
3. Validation through Simulation Experiments: Through a series of simulation experiments, the efficacy of the proposed scheme is substantiated. The results reveal that the scheme adeptly incorporates potential requestors into multicast clusters, amplifies the user count within said clusters, and adeptly addresses diverse user preferences for video quality. Consequently, this substantiates a notable enhancement in service quality.

The subsequent sections of this paper are structured as follows: Section 2 presents an overview of pertinent research endeavors, Section 3 furnishes an intricate exposition and modeling of the system architecture, Section 4 offers an in-depth analysis conducted through simulations, and Section 5 encapsulates the research outcomes in a concise summary.

## 2. Related Work

### 2.1. D2D Communication in the IoT

The adept deployment of D2D communication technology holds the potential to substantially enhance spectrum efficiency, alleviate the workload on base stations, and expand the communication scope for users. This culminates in the establishment of more supple and efficacious mobile communication networks. Nonetheless, owing to the finite availability of channel resources, instances arise where cellular users have fully utilized all accessible spectrums. Consequently, D2D users are compelled to engage in communication by repurposing the spectrum already utilized by cellular users.

In tackling the challenges presented by extensive data transmission scenarios, current research predominantly revolves around methodologies pertaining to resource allocation and computation offloading [5]. These strategies are geared toward optimizing the allocation of crucial resources, including spectrum and computational tasks, with the overarching objective of catering to the diverse user demands and judiciously harnessing the finite channel resources available within the IoT landscape. By harnessing sophisticated data processing techniques, researchers are able to effectively analyze the voluminous data generated by IoT devices, discern valuable insights, and make informed decisions to enhance the efficacy and efficiency of D2D communication. The assimilation of advanced big data processing techniques into D2D communication research paves promising pathways toward attaining heightened data transmission efficiency and superior user experiences within the ambit of D2D communication applications.

Cai et al. [6] delve into the resource allocation quandary within a context featuring a significantly larger cohort of D2D users in comparison to cellular users. Their primary objective is to maximize the overall system capacity. The authors cast the resource allocation challenge as a mixed-integer nonlinear programming conundrum [7], subsequently proffering a heuristic resource allocation method centered around graph coloring. In a bid to alleviate interference emanating from D2D connections, they introduce a D2D activation strategy anchored in protection zones [8]. In situations involving resource-constrained edge devices, where heterogeneous resource migration is necessitated, cooperative service migration frameworks are conceptualized in [9,10]. These frameworks tap into reinforcement learning techniques to optimize the interplay between service performance and associated costs. Additionally, the literature encompasses resource allocation strategies grounded in graph theory [11] and stochastic game formulations [12].

All the previously cited studies concentrate on centralized resource allocation algorithms, which yield favorable outcomes in resource allocation. However, these algorithms necessitate the base station to possess comprehensive user information, consequently engendering resource contention among users [13]. This, in turn, leads to intricate computational processes. In response to these challenges [14], we employ interruption probability and average achievable rates to ascertain optimal spectrum and power allocation for multiple D2D pairs amidst interference-prone scenarios. Ref. [15] introduces a groundbreaking hybrid transmission mode selection technique hinging on online reinforcement learning, attaining an equilibrium between broadband performance and resource utilization efficiency. Furthermore, Ref. [16] puts forth an unsupervised machine learning-driven hybrid intelligent clustering strategy alongside a Q-learning-rooted joint resource allocation and power control algorithm. These efforts culminate in augmented energy efficiency, which is gauged through throughput per unit energy consumption.

Nevertheless, D2D multicast encounters distinct challenges when juxtaposed with unicast transmission. The determinants influencing multicast group throughput encompass not solely channel resource allocation and power distribution [17] but also extend to considerations such as the count of multicast group users and the selection of cluster heads. Consequently, delving into multicast research and formulating effective multicast schemes consistently prove to be more intricate endeavors.

Diverse factors, encompassing physical locations, preferences, and social attributes, wield significant influence over the realms of edge computing and content caching [18]. In the literature [19], an exhaustive analysis was undertaken, leveraging copious datasets spanning physical locations and social attributes. This scrutiny aimed to dissect the impact of cluster head user selection on the configuration and performance of D2D multicast groups. Delving into social attributes between devices, [20] holds promise for ameliorating service quality during communication among users, concurrently curbing interference within vehicular contexts [21,22]. Within the framework of the literature [23], a D2D multicast scheme was propounded. Here, nodes congregate into D2D multicast groups grounded in physical attributes and social relationships. This approach not only culminates in the establishment of robust multicast clusters but also fosters a network underpinning social relationships. Transitioning to a distributed context, the literature [24,25] harnessed queuing theory and machine learning to configure the communication task offloading mechanisms, facilitating the transmission of substantial data volumes among diverse fog nodes within intelligent transportation settings. 

In the domain of enhancing D2D relayed multicast data packet reliability, network coding principles were harnessed, as evidenced by the literature [26]. Reinforcement learning-based methodologies have emerged as pivotal components in resource allocation solutions [27]. Pioneering a distinctive trajectory, a novel methodology grounded in multi-agent imitation learning for drone deployment was unveiled [28,29], proactively maximizing profits and user utilities within shared domains. To ensure the quality of user services and adeptly manage interference among users, Yuan [30] put forth a distributed multi-agent deep Q-network algorithm complemented by a partial information-sharing strategy. This paradigm empowers D2D agents to acquire the expertise needed to judiciously select optimal modes and channels. In parallel, within the framework of the literature [31], device location intelligence was harnessed to inform channel allocation strategies, targeting the maximization of overall effective throughput within multicast groups.

Currently, the majority of research in the realm of D2D communication tends to emphasize the impact of physical proximity, often allocating comparatively less attention to the inherent social attributes of users. Furthermore, investigations that seamlessly integrate D2D communication with video transmission and encoding are conspicuously lacking.

### 2.2. Scalable Video Transmission

D2D communication is marked by a diverse array of user terminal devices and channels, thereby posing challenges for conventional video coding methodologies to effectively accommodate the constantly evolving and intricate network milieu. Nevertheless, scalable video coding has emerged as a propitious remedy, showcasing notable versatility across network terminals and channels. This approach has garnered considerable attention in recent times, particularly within the ambit of D2D communication. Notably, in scenarios demanding substantial data computation and offloading, the role of scalable video coding technology is paramount.

Scalable video coding attains its adaptability by partitioning the video bitstream into multiple layers with distinct truncation points. A contemporary area of concentrated research revolves around the judicious selection of suitable bitstreams for video transmission, with the ultimate goal of furnishing users with elevated service quality. Present investigations concerning bitstream selection frequently center on prioritizing the transmission of the foundational video layer, underscoring the significance of these bitstream segments.

Furthermore, within the domain of D2D communication, scholars have delved into the intricacies of resource allocation pertaining to data transmission. This realm of inquiry encompasses an array of topics, including wireless channel allocation [32], the selection of modulation and coding schemes [33], resource allocation in both frequency and time domains [34], and power control strategies [35]. It is worth noting, however, that the complexity inherent in these resource allocation quandaries often renders them NP-hard, necessitating the application of sophisticated algorithms. For instance, in the case of Reference [32], cooperative game theory is harnessed to achieve efficient wireless channel resource allocation, while Reference [35] capitalizes on dynamic programming techniques to attain optimal solutions. Moreover, it is important to note that the current body of literature on D2D communication for video transmission predominantly revolves around video caching and delivery. For instance, researchers such as Wu et al. [36], Zhang et al. [37], and Li et al. [38] primarily focus on enhancing video transmission quality among users through the design of video caching schemes and collaborative sharing strategies. However, these approaches often fall short of comprehensively addressing the multifaceted factors present in real-world scenarios, including user preferences, user social attributes, and channel quality. 

Despite the potential advantages, research on D2D transmission with scalable video in the IoT remains somewhat limited. The user access rate has yet to attain its maximal capacity, thereby indicating that certain extant algorithms may fall short of adequately fulfilling the rigorous demands inherent in scalable video transmission within the IoT settings. Confronting these challenges and devising innovative remedies assumes paramount significance in order to fully unleash the inherent potential of scalable video coding within the domain of IoT applications.

## 3. System Model

In conventional cellular communication systems, multiple users with similar demands within a shared cell are compelled to access desired content independently via cellular links originating from the base station. Consequently, when multiple users initiate service requests, the base station is obligated to transmit identical content through distinct links. This transmission methodology is manifestly inefficient, resulting in a substantial redundancy in the transmission workload for the base station. Moreover, as the popularity of the transmitted content escalates, the redundancy amplifies correspondingly. To ameliorate redundant transmissions and enhance the efficiency in utilizing system resources, D2D communication presents significant potential for facilitating multicast transmissions. As a result, the exploration of D2D multicast communication has garnered extensive attention in recent years.

Nonetheless, owing to the substantial user influx accessing videos across distinct time segments, prevailing D2D multicast communication technologies can still engender pronounced instances of redundant transmissions, particularly concerning widely popular video content within smart cities or intelligent transportation contexts. Within the confines of the same geographical region, identical video content may be repetitively transmitted during various instances, thereby fostering considerable redundancy across the system. In an endeavor to further mitigate data redundancy and elevate the resource utilization efficiency inherent in D2D multicast communication systems, this research introduces a novel scalable video multicast scheme predicated upon video pushing. This scheme not only dispenses videos to users making specific requests but also proactively delivers videos to potential recipients, thereby augmenting the cohort of multicast recipients. This augmentation culminates in heightened throughput, ultimately leading to enhancements in spectrum utilization and energy efficiency.

Within a cellular small cell context, an array of IoT devices coexist, each harboring its distinctive video preferences. Furthermore, owing to device constraints and user inclinations, dissimilar individuals manifest varying expectations concerning video quality. When multiple users express a desire to partake in the viewing of identical video content, an opportunity emerges for the establishment of a potential D2D multicast cluster. Within this configuration, a cluster head is designated to take delivery of the video content from the base station, subsequently diffusing the multicast-shared video content to the remaining users within the cluster via D2D links. This procedure engenders a solitary cellular link at the base station while concurrently satiating the multimedia needs of multiple users, thereby considerably alleviating the operational burden imposed upon the base station.

However, owing to the divergent video requisites exhibited by individual users, the application of scalable video coding comes to the fore. The video content is consequently disseminated through the mechanism of layered multicast. This approach empowers users to selectively receive distinct video layers commensurate with their personalized preferences. The strategic utilization of scalable video coding, in conjunction with layered multicast methodologies, adeptly attends to the heterogeneous user requirements, culminating in an optimization of resource allocation within the expansive milieu of IoT big data analysis.

D2D users communicate by sharing spectrum resources with cellular users, which leads to co-channel interference. To ensure that all users within a multicast cluster can receive multicast data properly, it is necessary to guarantee that the user with the worst channel quality can receive multicast data successfully. Let us denote the multicast-receiving user with the worst channel quality. According to Shannon’s formula, the channel capacity, which represents the upper limit of the multicast transmission rate for the multicast group, is given by:

(1) $$R_{\min}=B\log_{2}\left(1+\frac{p_{h}g_{hm}}{I_{cm}+N_{0}}\right),$$

where $p_{h}$ represents the transmit power of the cluster head node $u_{h}$, B represents the channel bandwidth, $N_{0}$ represents the Gaussian channel noise, $I_{cm}$ represents the interference from cellular users received by the user $u_{m}$, and $g_{hm}$ represents the channel gain from the cluster head $u_{h}$ to the receiving user $u_{m}$.

In the network, when transmitting scalable videos, different numbers of video layers require different bitrates, represented by the set $\left\{r_{1},r_{2}\cdots r_{k}\right\}$. Each user has unique video quality requirements, corresponding to receiving a different number of video layers in scalable videos. Hence, each user i has distinct bitrate demands, denoted as $R_{i}^{dem}$. Users are not interested in receiving video streams that exceed their requirements and will not receive them. However, they will make efforts to receive the portions that do not exceed their needs. We introduce a function:

(2) $$\delta_{i}\left(A\right)=\{\begin{array}{ll} A & \left(A<R_{i}^{dem}\right)\\ R_{i}^{dem} & \left(A\geq R_{i}^{dem}\right) \end{array}$$

to represent the relationship between the network’s bitrate and the bitrate received by users. The total bitstream received by all users within a multicast cluster, including the cluster head, is denoted as:

(3) $$R_{total}=\sum_{i=1}^{N}\rho_{i}\delta_{h}\left\{\delta_{i}\left[B\log_{2}\left(1+\frac{p_{h}g_{hm}}{I_{cm}+N_{0}}\right)\right]\right\},$$

where $\rho_{i}$ indicates whether the user i joins the multicast group, taking values of 0 or 1, where 1 indicates joining the multicast group and 0 indicates not joining. N represents the total number of users. It can be observed that there are three factors influencing the total bitstream received in the D2D multicast transmission system: cluster head selection, the number of receiving users, and the minimum channel capacity of receiving users. Optimal cluster head selection and an increase in the number of receiving users within the multicast cluster should be pursued to enhance the energy efficiency and spectral efficiency of the multicast system.

In prevailing configurations of D2D multicast video delivery systems, a predominant majority of them employ non-scalable video coding techniques, and they neglect to address the variability in video quality requisites among distinct users. Consequently, these systems are constrained to providing a uniform video quality level for all users. Furthermore, certain investigations have not fully accommodated the temporal disparities inherent in users’ video requests; this pertains to scenarios where users soliciting the same video content might do so at disparate time instances. In the context of popular videos, it is noteworthy that video requests originating from users within the same geographical vicinity may transpire across multiple time intervals. This phenomenon necessitates repeated retrieval of the video content from the base station and potentially gives rise to numerous instances of D2D multicast clusters. Evidently, the current state of traditional D2D multicast video delivery systems exhibits appreciable scope for refinement, with the potential to significantly augment transmission efficiency.

**Figure 1:**
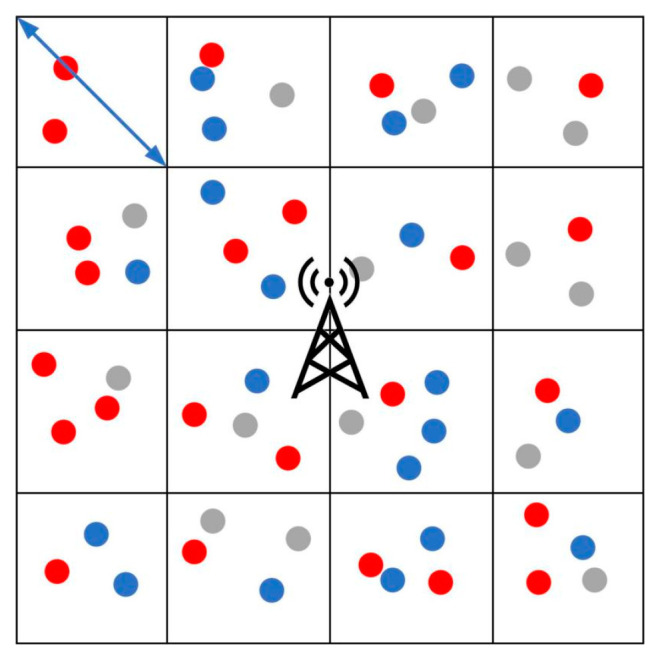
Cluster-based methodology using a grid-based approach.

In this investigation, users expressing a demand for video content are classified into two distinct categories. The first category encompasses users who instigate video requests in the present moment, and they are termed “service request users.” The second category comprises users who, although not initiating video requests in the current timeframe, share an identical video demand; these are denoted as “potential request users”. As delineated in Figure 1, service request users are visually depicted in red, potential request users are denoted in blue, and users devoid of video demands are indicated in gray.

The entire cellular expanse is partitioned into multiple grids using the approach expounded in Reference [39]. In these grids, any two users falling within the same grid exhibit an inter-user distance lesser than the designated maximum communication range for D2D interactions, thereby warranting the feasibility of D2D communication. Within a conventional D2D multicast system, service request users sharing a common grid congregate to form a D2D multicast cluster, wherein one user is elected as the cluster head to oversee the distribution of data. However, the conventional multicast distribution strategies have not optimally harnessed the inherent advantages intrinsic to D2D multicast communication. Consequently, a notable potential remains untapped in terms of bolstering the count of users participating within the D2D multicast cluster.

If videos could be multicast concurrently to both service request users and potential request users who express a willingness to receive them, the aggregate count of multicast recipients would witness a substantial augmentation. This, in turn, would yield a pronounced enhancement in the energy and spectrum efficiency of the entire system. While a judicious video push service undoubtedly contributes to an elevated user experience, a caveat emerges—frequent dissemination of undesired videos to users may impede their overall experience.

Given the enigma posed by the elusive nature of potential request users, a universal video service push to all users is rendered unfeasible. Consequently, the imperative to winnow out potential request users assumes prominence. This filtration process engenders the selective delivery of videos solely to the identified, filtered potential request users. These filtered potential request users, alongside the service request users who consent to the video push, collectively constitute the pivotal constituents constituting the D2D multicast cluster. Subsequently, the subsequent phase entails the meticulous appointment of an apt cluster head tasked with the retrieval of the video content from the base station. This cluster head subsequently orchestrates the distribution of the video content among the remaining users encompassed within the multicast cluster. Within this research endeavor, our focus is twofold: firstly, the precision prediction of potential request users to participate within the multicast cluster, and secondly, the discerning selection of an optimal cluster head. This selection process aims to establish an expanded, more steadfast multicast cluster characterized by heightened capacity.

### 3.1. User Demand Perception and Video Push

Users sharing the same video demand often exhibit disparate temporal patterns in initiating service requests. Propagating videos to potential request users offers the potential to facilitate simultaneous video reception for a greater number of users, thereby amplifying multicast efficiency. However, an overabundance of video pushes may introduce a degradation in user experience. Several scenarios can hinder successful video pushes, encompassing instances where a device is marred by insufficient battery power or storage capacity to accommodate video reception. Furthermore, user preferences must be heeded, as certain users may opt not to receive video pushes to avoid disruptions.

Furthermore, the success of a video push hinges upon a confluence of factors. Even if a user and their device satisfy the prerequisites for video reception, the pertinence of the pushed video content to the user’s interests remains pivotal. Indeed, a video push can adversely affect the quality of a user’s service experience if the pushed video content lacks relevance, thus rendering the entire video push service inconsequential. In light of these considerations, this section undertakes a comprehensive analysis of potential request users’ demands. The objective is to gauge their inclination to embrace video pushes and, by extension, ensure the attainment of a satisfactory user experience.

#### 3.1.1. Probability of Video Requests

The likelihood of a user soliciting a specific video hinges on two pivotal factors: the video’s overall popularity and the user’s distinct personal interests. Consequently, in the computation of the video request probability, it becomes imperative to account for both the aggregate attributes characterizing videos within the IoT context and the idiosyncratic predilections of individual users. This holistic approach is essential to engender outcomes that faithfully encapsulate the true video request probability.

1. User Interest

The user’s past video-viewing behavior serves as a reflection of their individual interests. If a user has engaged with a specific genre of video content on numerous occasions within a recent timeframe, it serves as an indicative manifestation of their affinity toward that particular video genre. The frequency with which a distinct video genre is accessed and viewed by a user can be deemed a reliable gauge of their degree of interest. As such, the proportion of video content consumption attributed to a specific genre by an individual user can be mathematically expressed as follows:

(4) $$f_{i,l}=\frac{x_{i,l}}{x_{i}},$$

where $x_{i,l}$ represents the number of times the user $u_{i}$ has watched videos of type l and $x_{i}$ represents the total number of videos watched by the user $u_{i}$. Nonetheless, the equivalence in video count across different categories might not prevail, potentially leading to a skewed request rate for certain video categories due to their lower quantity. Furthermore, dissimilarities in video durations emerge as another confounding factor. For instance, succinct video clips could span a mere few seconds, while full-length movies may extend over several hours. Consequently, an elevated number of clicks on brief videos juxtaposed with a comparatively lower count for movies does not necessarily signify a predilection for one over the other. To redress these complexities and attenuate their influence, an alternative approach entails gauging the proportion of videos consumed within each category from a distinct standpoint. Equation (4) assumes uniform weighting for each viewed video concerning user interest. Alternatively, we can counteract the disparities in video duration and quantity by positing an equitable aggregate weight for all clicks within each video category. The quantification of a user’s video consumption within a specific category can be articulated as follows:

(5) $$f_{i,l}^{*}=\frac{x_{i,l}}{x_{l}}/(\sum_{l=1}^{L}\frac{x_{i,l}}{x_{l}}),$$

where $x_{l}$ represents all user requests for the l-th video type and L denotes the total number of video types. To precisely gauge the extent of user interest in a specific video category, we can integrate the two aforementioned formulas to derive the following expression:

(6) $$F_{i,l}^{interest}=\eta \frac{x_{i,l}}{x_{i}}+\left(1-\eta \right)(\frac{x_{i,l}}{x_{l}}/\sum_{l=1}^{L}\frac{x_{i,l}}{x_{l}}).$$

In the formula, η represents the weight coefficient. Some users frequently search for videos of interest. However, users who have not watched a particular video may not be able to determine whether they like its content. As a result, users may click on videos that they dislike or have no interest in. When performing calculations, it is necessary to establish a threshold to filter out this portion of requested information. Assuming the threshold is denoted as $\xi_{th}$, each historical viewing record included in Equation (5) should satisfy the condition “$t_{i,l}^{j}/T_{i,l}^{j}>\xi_{th}$,” where $t_{i,l}^{j}$ represents the duration of a historical record and $T_{i,l}^{j}$ represents the total duration of the corresponding requested video. If the duration of a historical record is too short, it cannot reflect the user’s interest in that video, and it will not be considered when calculating the level of interest.

2. Video Popularity

A significant body of research has demonstrated that while the specific video content may differ across regions, user requests for video content within the same region adhere strongly to the Zipf distribution. Therefore, the popularity of videos is commonly computed using the Zipf distribution. Let set “$V=\left\{v_{1},v_{2}\cdots v_{M}\right\}$” represent the collection of video requests made by users in a particular local area during a defined time period, with subscripts denoting their respective popularity rankings. Accordingly, the popularity of any video “$v_{m}$” within this set can be represented as follows:

(7) $$F_{m}^{pop}=\frac{m^{-\gamma }}{\sum_{c=1}^{M}c^{-\gamma }},1\leq m\leq M,$$

where γ represents the Zipf distribution exponent specific to the locality, which can be referred to as the video popularity factor. A greater numerical magnitude denotes a swifter wane in popularity, implying that a select few highly popular videos amass a disproportionate share of views. Within the context of device conditions and user inclinations, the likelihood of a user being presented with a video recommendation is intricately linked to two principal factors: the video’s prevalence and the user’s degree of interest in said video. Therefore, the level of interest of the user u_i_ in a video $v_{m}$ can be expressed as:

(8) $$P_{i,m}^{interest}=\alpha F_{m}^{pop}+\left(1-\alpha \right)F_{i,l}^{interest},$$

where α represents the weight coefficient and l denotes the category code of video $v_{m}$.

#### 3.1.2. User States

The prior calculation of the probability of users accepting push notifications did not encompass the current status and device conditions of the users. Within a given user’s context, their receptiveness to video push notifications exhibits temporal fluctuations. To elucidate, users might refrain from disturbances during work or study periods, while embracing such notifications during leisure or relaxation intervals. Moreover, the state of users’ devices also assumes significance, with the reception of videos necessitating specific physical prerequisites. Initially, the establishment of a reliable link between the user receiving the video and the cluster head is imperative. Furthermore, the act of video content reception entails an expenditure of energy.

As a result, users have two distinct states: the state of accepting push notifications and the state of rejecting push notifications. Users can actively set these two states. They set the state to accept push notifications when it is appropriate for them to receive videos, and they set the state to reject push notifications during time periods when it is not suitable to receive videos. The device can perceive its current state and automatically determine if it meets the physical conditions for receiving video push notifications. For example, it checks if the current battery level is sufficient to fulfill the requirements for receiving videos and if there are physical conditions to establish a stable D2D link. When users set the state of accepting push notifications, if the device senses that its current state is insufficient to receive video push notifications, the device automatically modifies its current state. Let $\varphi_{i}$ represent the user’s ($u_{i}$) state, taking values 0 or 1, where 0 represents the state of accepting push notifications and 1 represents the state of rejecting push notifications. When calculating the probability for users to accept video push notifications, we only need to consider users who are in the state of accepting push notifications. For users in the state of rejecting push notifications, their probability of accepting push notifications is 0. Taking into account these two states, the willingness of the user $u_{i}$ to accept video $v_{m}$ push notifications can be represented as:

(9) $$P_{i,m}^{acc}=\varphi_{i}P_{i,m}^{interest}.$$

#### 3.1.3. User Demand-Aware Push Algorithm

In the preceding section, this paper computed the likelihood of users in the “accepting push notifications” state embracing a specific video push. This computation considered both the user’s viewing history and the video’s popularity. The objective of the video push service is to elevate user experience quality while refraining from pushing videos to users who have opted to reject push notifications. In scenarios involving users who are amenable to video push notifications, a judicious approach is employed, wherein video pushes are administered selectively. This strategic selection aims to curtail superfluous resource consumption and minimize potential disturbances. For any given user subjected to a push notification, their predisposition to accept video pushes must conform to the following criterion:

(10) $$P_{i,m}^{acc}>P_{th}^{acc},$$

where $P_{th}^{acc}$ represents the minimum threshold of willingness for a user in the state of accepting push notifications to accept a specific video push. If a user’s willingness to accept a particular push is lower than this threshold, pushing that video to the user may diminish their service experience quality. In summary, when a service request user initiates a video request, the system should push videos to users in the state of accepting push notifications based on their willingness to accept video pushes and construct multicast clusters.

### 3.2. Cluster Head Selection Mechanism

#### 3.2.1. Social Relationship Perception

Given the limited scope of D2D communication and the dynamic movement patterns of D2D users, ensuring the persistence of the wireless channel state for seamless D2D communication presents a notable challenge. This challenge inherently raises the prospect of potential disruptions in D2D links. The efficacy of a D2D communication system is intrinsically tied to the stability of its D2D links. Extensive research has substantiated the intrinsic correlation between D2D communication stability and users’ social interactions.

**Figure 2:**
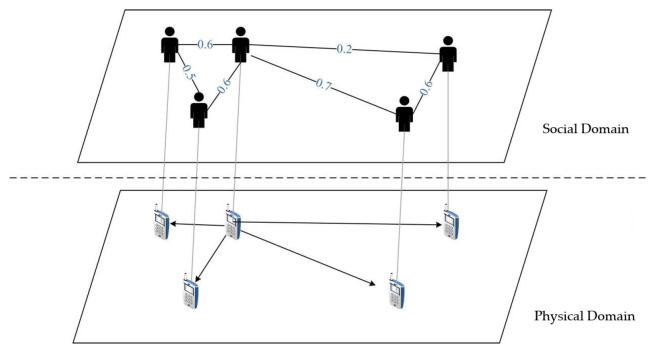
Physical and social domains among end devices.

Several attributes serve as indicative markers of user social relationships, including trust, mobility congruence, and shared interests. As depicted in Figure 2, the illustration underscores the interplay between the physical domain (representing tangible communication conditions) and the social domain (depicting social connections) among terminal entities. Notably, the establishment of a resilient wireless link necessitates the convergence of both physical communication prerequisites and robust social affiliations.

Extensive research has been conducted in the domain of social relationships. In this study, a user preference analysis approach is adopted to gauge the intensity of social connections between two users situated within the same grid. As previously elucidated, users’ receptiveness to widely favored videos serves as a credible proxy for gauging their preferences. The formulation presented in Equation (6) facilitates the comprehension of a user’s proclivity toward various video categories, thereby enabling the derivation of the user’s interest preference vector. Specifically, for a given user denoted as $u_{i}$, their corresponding interest preference vector is designated as:

(11) $$P_{i}^{interest}=\left\{P_{i,1}^{acc},P_{i,2}^{acc}\cdots P_{i,M}^{acc}\right\}.$$

Next, the social relationship strength between two users is calculated using cosine similarity. The social relationship strength between user $u_{i}$ and user $u_{j}$ is represented as:

(12) $$S_{i,j}=\frac{\sum_{l=1}^{L}P_{i,l}^{acc}P_{j,l}^{acc}}{\sqrt{\sum_{l=1}^{L}{\left(P_{i,l}^{acc}\right)}^{2}}\sqrt{\sum_{l=1}^{L}{\left(P_{j,l}^{acc}\right)}^{2}}}.$$

Formula (12) enables the computation of the social relationship strength between any two users.

#### 3.2.2. Cluster Head Selection Algorithm

In the preceding section, the inclination of users in the state of accepting push notifications to embrace a specific video push was deduced through an amalgamation of their viewing history and video popularity. Following this, the act of video pushing transpired based on users’ proclivity, with these recipients being amalgamated alongside service request users to forge cohesive D2D multicast clusters. Within the context of D2D multicast transmission, the meticulous curation of cluster heads emerges as a pivotal determinant, wielding pronounced influence over multicast cluster performance. In light of this, the formulation of a cluster head selection framework becomes imperative and is aimed at discerning an apt cluster head and configuring a multicast infrastructure characterized by elevated transmission capacity and steadfast stability.

**Figure 3:**
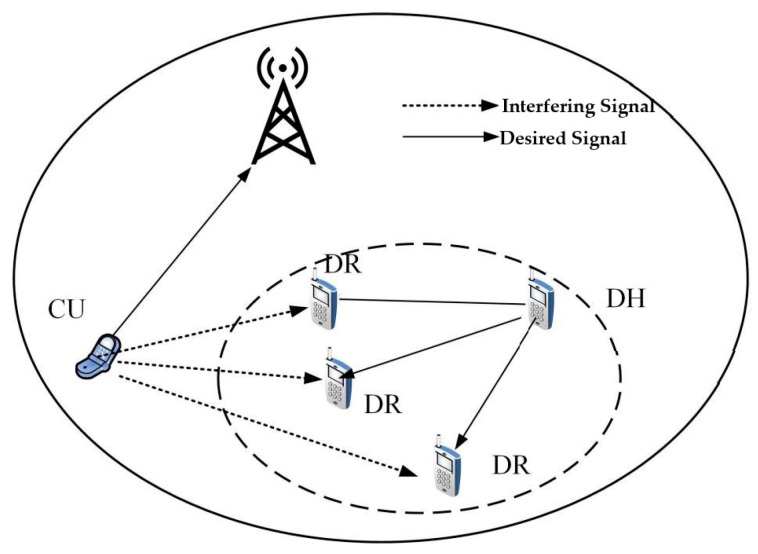
Illustration of interference in D2D multicast communication.

A multicast cluster is preemptively established through proactive video broadcasting, with the designated user ensemble of the multicast cluster delineated as $u_{c}$. D2D multicast communication strategically harnesses the uplink resources of cellular users, thereby yielding a shared milieu of spectrum resources underscored by co-channel interference. As illustrated in Figure 3, the nomenclature CU pertains to cellular users, DR signifies the receiving cohort within D2D communication, and DH denotes the steward of the D2D multicast cluster. Assuming that the mantle of multicast cluster leadership is conferred upon the user $u_{j}$, when both the multicast cluster overseer and the cellular user emit signals under powers $P_{j}$ and $P_{c}$, correspondingly, the channel capacity applicable to any user $u_{i}$ ensconced within the multicast cluster’s receptive assemblage is formulized as:

(13) $$R_{j,i}=B\log_{2}\left(1+\frac{P_{j}g_{j,i}}{P_{c}g_{c,i}+N_{0}}\right).$$

In this representation, $g_{j,i}$ denotes the channel gain between the cluster head $u_{j}$ and the D2D receiving user $u_{i}$, $g_{c,i}$ represents the channel gain between the cellular user $u_{c}$ and D2D receiving user $u_{i}$, and $N_{0}$ is the Gaussian noise with zero mean. Any user in the multicast cluster can be selected as the cluster head or receiving user. Due to the varying distances and channel qualities between any two users, the channel capacity between the cluster head and other users differs when different users are selected as the cluster head. $R_{j}^{\min}$ represents the minimum channel capacity between the user $u_{j}$ and all other users in the multicast cluster. If the user $u_{j}$ is chosen as the cluster head, the maximum multicast rate for the cluster head is denoted as $R_{j}^{\min}$. Each user has different video quality requirements and needs to receive a different number of video layers, resulting in varying bitrate demands for each user. Users are not interested in or receive video streams beyond their own needs. When the user $u_{j}$ is the cluster head, firstly, the user $u_{j}$ obtains the required video content through the base station, fulfilling their own video demands. Then, scalable video layered multicast is performed. In order to guarantee the real-time decoding and seamless viewing of the video by receiving users, it is imperative that the bitrate of the multicast video layers remains below the prescribed multicast rate threshold. Failure to adhere to this constraint may engender frequent buffering and disruptive stuttering. Subsequently, the user entrusted with the role of cluster head undertakes the task of multicasting the obtained video content at a rate of:

(14) $$R_{j}^{send}=\delta_{j}\left(R_{j}^{\min}\right).$$

while the multicast-receiving users selectively receive based on their demands at a rate of:

(15) $$R_{j,i}^{receieve}=\delta_{i}\left[\delta_{j}\left(R_{j}^{\min}\right)\right].$$

The efficacy of multicast transmission is influenced not solely by the physical characteristics of users but also by their social attributes. A more robust social connection between two users tends to correlate with improved stability of the D2D link. To provide an accurate gauge of the transmission performance within the D2D multicast system, the utility metric $R_{j}^{utility\;}$ is introduced. This metric takes both channel quality and the robustness of social relationships between users into account. The formulation of this utility value is represented as:

(16) $$R_{j}^{utility\;}=\sum_{i=1}^{N}\rho_{j}S_{j,i}\delta_{i}\left\{\delta_{j}\left[R_{j}^{\min}\right]\right\}.$$

where $S_{i,j}$ represents the social relationship strength between the cluster head user $u_{j}$ and receiving user $u_{i}$ The utility value $R_{j}^{utility\;}$ simultaneously considers the physical information of the users and social relationship information, making it a more practical communication environment indicator than traditional throughput metrics.

## 4. Performance Evaluations

The proposed approach outlined in this paper is validated through extensive simulations carried out on the MATLAB platform. The purpose of these simulations is to provide a comprehensive evaluation of the clustering and cluster head selection methodology put forth in this study. The performance of the algorithm is rigorously assessed across various communication scenarios to ensure a robust analysis.

In order to evaluate the effectiveness of the proposed clustering and cluster head selection scheme, a series of simulation experiments are conducted. These experiments involve comparing the algorithm’s performance against alternative schemes operating within the same communication environments. The benchmarked schemes encompass social-aware strategies, interference-aware methodologies, and random selection approaches. The social-aware scheme is designed to establish D2D multicast clusters by taking into account nearby service request users, with the cluster head chosen based on the user possessing the highest number of stable social relationship links. Similarly, the interference-aware scheme constructs D2D multicast clusters considering nearby service request users, and the cluster head selection is based on the user with the most stable links. By conducting these simulation experiments and comprehensive comparisons, the efficacy of the proposed scheme is thoroughly assessed, ensuring a robust evaluation of its performance across a variety of communication contexts.

Given the central focus of this paper on enhancing the quality of video transmission, the scope of short-range video transmission is confined to small-scale D2D communication scenarios. Within the purview of such small-scale D2D communication contexts, the fundamental objective of D2D communication is the direct exchange of data among devices that are situated in relatively close proximity. This encompasses a spectrum of activities including social interactions, file sharing, and gaming. In these scenarios, the communication range typically spans from several tens to a few hundred meters. For the purpose of our simulations, the maximum D2D communication radius is set at 60 m. Given that specific values for D2D communication simulation parameters are not explicitly defined, we have drawn inspiration from the general parameters for D2D communication simulations, as outlined in Reference [36]. The concrete selection of experimental parameters is detailed in Table 1 above.

The simulation environment is structured as a single-cell model, with users distributed randomly within a grid. The geographic positions of users adhere to a homogeneous Poisson distribution. Additionally, key factors pertaining to user count in the context of small-scale communication are integrated into the fixed parameters of the simulation environment. The specifics of the communication parameters are outlined as follows: video bitrates ranging from 600 kbps to 3000 kbps are partitioned into five distinct layers, with each layer necessitating a constant bitrate of 600 kbps. Each successive layer corresponds to an increment in bitrate, denoting a varying level of video quality. Unless explicitly indicated otherwise in subsequent simulations, it is assumed that the likelihood of user requests for each level of video quality is as follows: 0.01, 0.02, 0.05, 0.1, and 0.79. Furthermore, users engaged in D2D communication opportunistically share uplink cellular channel resources alongside cellular users. The channel model encompasses factors such as path loss, Rayleigh fading, and Gaussian channel noise. The core configurations of the primary parameters are itemized in Table 1. The values indicated therein, serving as default settings, are applicable to any experimental scenario unless expressly stipulated otherwise.

Multicast performance is influenced by a range of factors, which can be fundamentally categorized into two main groups: the number of users within the multicast group and the choice of the cluster head. The subsequent sections of this paper will delve into an analysis of the multicast scheme’s performance, specifically focusing on these two facets, through comprehensive simulations.

### 4.1. Impact of Potential Requesting User Count on Utility Value

The impact of potential request users is pivotal in assessing the efficacy of the recommendation algorithm proposed in this study. Comparative experiments were meticulously conducted across varying scenarios involving potential request users. This allowed for an exploration of the interplay between the algorithm’s performance and key parameter settings, encompassing factors such as willingness threshold, popularity factor, and fluctuations in video quantity. Notably, distinct configurations of potential service users yielded diverse system performances. Nonetheless, an overarching trend emerged, wherein an augmented count of potential service users correlated with heightened utility values within the D2D multicast system. This utility value serves as a reliable indicator of the average throughput achieved by the D2D multicast transmission system.

**Figure 4:**
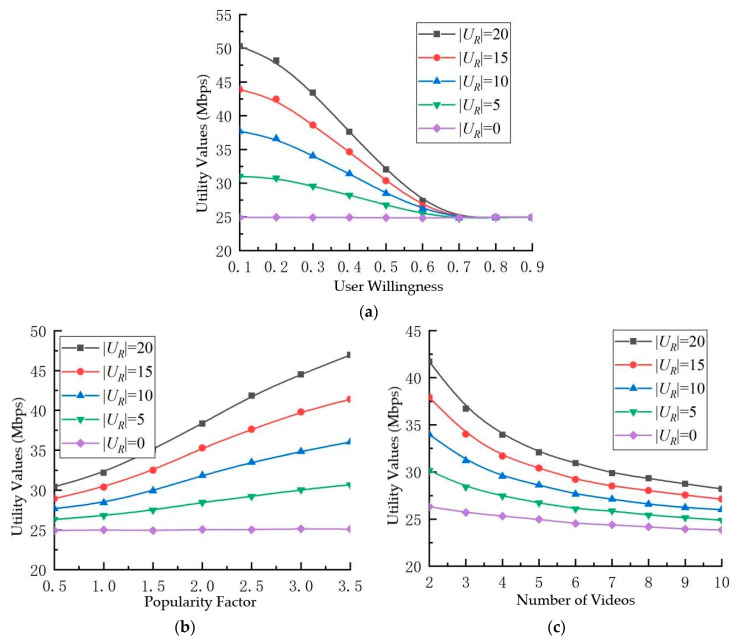
Influence of different parameters and potential requesting user count on utility value. (**a**) Utility values and user willingness to accept thresholds, (**b**) utility values and popularity factors, (**c**) utility values and number of videos.

As shown in Figure 4a, there is a clear downward trend in the utility value as the willingness threshold $P_{th}^{acc}$ for accepting video recommendations increases. |UR| represents the number of potential requesting users, and as the number of potential requesting users increases, the utility value also increases. However, as the willingness threshold $P_{th}^{acc}$ increases, the utility value decreases at a faster rate. Once the willingness threshold exceeds a certain range, the rate of decline in the utility value slows down. In the proposed video recommendation scheme, video recommendations are only sent to potential requesting users within the D2D multicast cluster if their willingness $P_{i,m}^{acc}$ to accept video recommendations is above a threshold $P_{th}^{acc}$, leading to their inclusion in the multicast cluster. With an escalation in the threshold value, the count of users satisfying the established condition exhibits a corresponding diminution, consequently leading to a reduction in the utility value. The observed diminishing trajectory in the simulation outcomes offers insights into the dispersion of users’ propensity to embrace video recommendations. Notably, a predominant proportion of users tend to possess willingness levels falling within the range of 0.2 to 0.7. The selection of the willingness threshold significantly influences users’ service encounters. An excessively low threshold gives rise to frequent disruptions in users’ activities, whereas an excessively high threshold culminates in the majority of users failing to meet the stipulated requisites for video recommendations. This, in turn, curtails the effectiveness of the proposed scheme’s performance.

Figure 4b illustrates the correlation between the utility value and the popularity factor (γ). As the popularity factor escalates, the depicted curve in the graph exhibits an ascending trajectory, which is indicative of an augmentation in the utility value. This phenomenon can be attributed to videos endowed with a heightened popularity factor, which undergoes a swifter waning in popularity over time. As a consequence, a select few highly popular videos garner an elevated number of clicks, thereby resonating with users’ predilections. Notably, during the process of video recommendations, users manifest an elevated propensity to accept the recommended content, subsequently fostering a greater influx of users into the multicast cluster. 

Figure 4c portrays the connection between the utility value and the number of videos (|F|). A discernible trend manifests where an escalation in the number of videos gives rise to a reduction in the utility value. This outcome stems from the proliferation of videos, engendering a relatively diminished level of popularity for each individual video. Consequently, the willingness of potential requesting users to embrace video recommendations undergoes a decline.

In synthesis, an augmented count of potential requesting users aligns with an amplified efficacy of the video recommendation algorithm proffered in this study. Moreover, the manipulation of distinct parameter configurations markedly influences the performance of the system.

### 4.2. Impact of Cluster Head Selection Options on Utility Values

The aforementioned simulation outcomes underscore the direct correlation between the count of potential requesting users and the population within the multicast cluster, thereby exerting a substantial influence on the multicast utility value. Subsequently, the efficacy of the cluster head selection scheme posited in this study is subjected to validation through ensuing simulation analyses.

**Figure 5:**
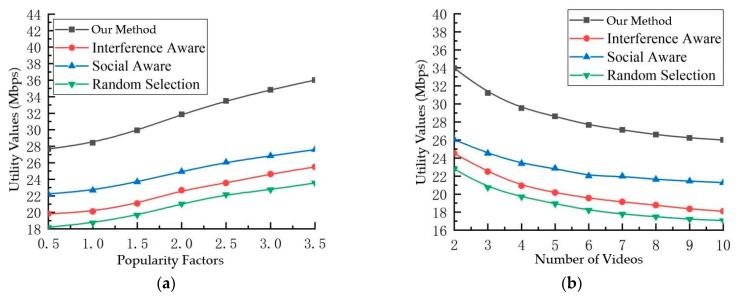
Effect of different parameters and scenarios on the degree of utility. (**a**) Utility values and popularity factors, (**b**) utility and number of videos.

Figure 5 presents a comparative analysis of diverse schemes across a spectrum of video quantities and popularity factors. The discernible trend indicates that the multicast scheme advocated in this paper outperforms its counterparts, with the social-aware scheme showcasing superior performance over the interference-aware scheme. Notably, the random selection scheme registers the least favorable performance. In general, a consistent pattern emerges across all schemes: the utility value ascends in response to heightened popularity factors, yet wanes with a proliferation in the number of videos.

The impact of distinct parameter configurations on the utility value of the multicast scheme has been deliberated from dual vantage points. Yet, an additional pivotal factor influencing the efficacy of the proposed scheme pertains to the diversity in users’ preferences for video quality. Throughout the simulation, videos have been stratified into five layers, thereby affording five discrete levels of video quality. The likelihood of a user expressing a preference for each quality tier is designated as $\left\{P_{1}^{\mathrm{layer}},P_{2}^{\mathrm{layer}},P_{3}^{\mathrm{layer}},P_{4}^{\mathrm{layer}},P_{5}^{\mathrm{layer}}\right\}$. The measure of variability in users’ predilections for video quality is succinctly encapsulated by the following variance:

(17) $$\Delta =\frac{\sum_{i=1}^{5}P_{i}^{layer}{\left[\left(\sum_{j=1}^{5}P_{j}^{layer}j/5\right)-i\right]}^{2}}{5}.$$

**Figure 6:**
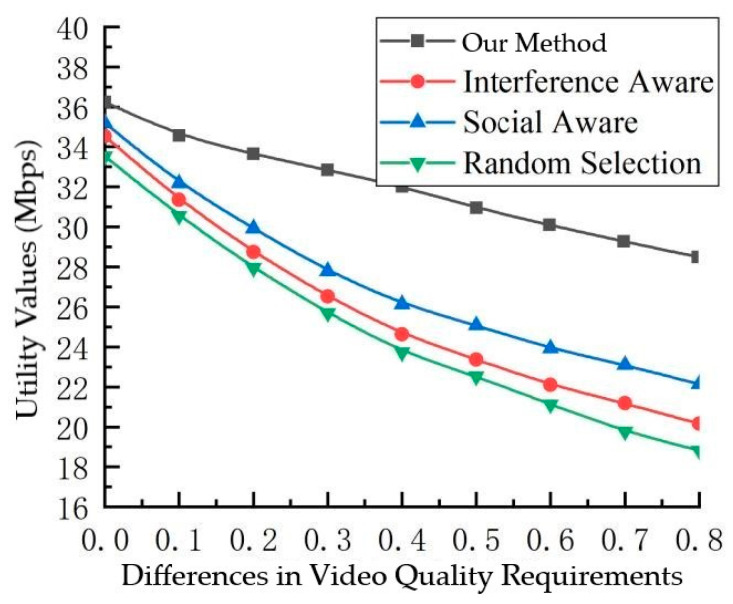
Relationship between utility values and variability in video quality requirements.

Figure 6 portrays the interplay between the utility value and the variance in users’ preferences for video quality. Here, a uniform mean bitrate of 1.8 Mbps is stipulated for all users, yet individual users exhibit divergent proclivities for video quality. As the spectrum of quality preferences widens, the utility value undergoes a gradual attenuation. Notably, the diminishing trend is less conspicuous in the case of the proposed algorithm, which is in contrast to the comparative algorithm, which exhibits a precipitous downturn. This nuanced distinction can be attributed to the comprehensive framework underpinning the proposed algorithm, which not only takes channel quality and social attributes into account but also caters to users’ distinct appetites for video quality. The proposed approach strategically favors users with higher quality expectations as cluster heads for video multicast forwarding. Conversely, the comparative algorithm evinces a proclivity to appoint users with lower-quality requirements as cluster heads. This inclination, driven by users’ inherent self-interest, engenders a predicament in meeting the multifaceted needs of the multicast cluster’s recipients.

## 5. Conclusions

The wireless network realm is inherently shackled by finite spectrum resources. In the ambit of transmitting copious video data, the pivotal quandary encountered by contemporary video transmission services resides in adroitly modeling and dissecting these confined spectrum assets. This vanguard research embarks upon the precincts of D2D multicast for scalable videos, proffering forth a scalable video multicast blueprint imbued with user demand data acumen. Commencing the journey, user predilections are meticulously cast and scrutinized, harnessing a trove of data encompassing their historical video consumption trajectory and the tapestry of video popularities. This compendium of insights coalesces to fathom users’ proclivity to embrace specific video recommendations, thus engendering an assessment of their amenability. In sequitur, videos are judiciously channeled toward users manifesting a pronounced predisposition, thereby augmenting the retinue of recipients within the multicast constellation. This endeavor is galvanized by an innovative clustering algorithm that ushers in a coterie of latent inquirers. Concomitantly, cognizant of users’ channel fidelity, social dynamics, and video quality predilections, a cluster head selection algorithm is meticulously contrived. This algorithm, in essence, choreographs a clustering architecture calibrated to distill maximal utility from the tapestry of D2D multicast. Culminating in an array of simulation experiments, the proposed paradigm adeptly infuses nascent inquirers into the multicast coterie, augments the phalanx of cluster constituents, resonates with an eclectic gamut of video quality requisites, and amplifies the echelons of service quality governing video transmission within the annals of D2D communication among an assemblage of devices.

## Figures and Tables

**Table 1:** Simulation parameters.

Parameters	Values
Maximum D2D Communication Radius	60 m
Channel Bandwidth (B)	180 KHz
Path Loss Factor (α)	4
$\mathrm{Noise}\;\mathrm{Power}\;\mathrm{Spectral}\;\mathrm{Density}\;(N_{0}$)	−174 dBm/Hz
$\mathrm{Cellular}\;\mathrm{User}\;\mathrm{Transmit}\;\mathrm{Power}\;(P_{c}$)	23 dBm
$D2D\;\mathrm{Transmit}\;\mathrm{Power}\;(P_{h}$)	23 dBm
$\mathrm{Willingness}\;\mathrm{Threshold}\;(P_{th}^{acc}$)	0.5
Number of Videos (|F|)	5
Video Popularity Weight (λ)	0.5
Number of Users Requesting Services	20

## Data Availability

No public datasets were used to support this study.
